# Supplementary material for: Maternal tobacco smoking and childhood obesity in South Africa: A cohort study
Source: PLoS One. 2023 Feb 9;18(2):e0268313. doi: 10.1371/journal.pone.0268313 (PMC9910632; doi:10.1371/journal.pone.0268313)
Supplement: Attachment — Submitted filename: comment for plose.docx [file pone.0268313.s001.docx]

Dear author, I attached some of my comments here.

-_You have to put line number for the whole part of your manuscript

Abstract

-You did not included introduction.

-The method part does not include important information like Sample size, sampling technique, study design, the major assumptions checked and the measures of association used.

Methods

-Why you put all portions of the method in one paragraph. It is better to put in different sub-headings.

-Although it is secondary data, for the reader of the paper to be clear you have to elaborate about the sampling method, the way you reach the sample size, data collection tools and procedures.

-What about the inclusion and exclusion criteria, data quality control and ethical assurance?

-You didn’t described about the models (what is included in model1, model2 and model3 and what criteria was used to compare those models and which one is better)

-What measure of association you had used?

Result

-Why did you prefer using mean and standard deviation?

-The variables did not have category

-The tables need full title, which describes about place, person, time and the problem. In addition, above the table you have to describe major things from the table using texts.

- In result section, Interpretation of the finding is mandatory

Discussion

-you did not included the possible justifications
