# Supplementary material for: Maternal tobacco smoking and childhood obesity in South Africa: A cohort study
Source: PLoS One. 2023 Feb 9;18(2):e0268313. doi: 10.1371/journal.pone.0268313 (PMC9910632; doi:10.1371/journal.pone.0268313)
Supplement: Attachment — Submitted filename: Revision report_maternal smoking and child health in SA.docx [file pone.0268313.s002.docx]

Revision report on the manuscript “**I**mpact of maternal tobacco smoking on childhood obesity in South Africa”

**Dear anonymous reviewers, thank you for your insightful comments and suggestions which have helped improve the quality of our manuscript. For each of the comments highlighted in yellow, the answer is provided, which outlines the suggested corrections and the references thereof. Your contribution is gratefully acknowledged.**

**Editor**

The topic of the research is important.

**Answer. Thank you**

The method section needs scientific reformulation and organization under subtitles,

**Answer. This has been addressed. The method section has been updated to “Materials and Methods” with the sub-headings “Data” and “Empirical strategy” and their respective contents updated accordingly. Please refer to pages 5 and 6 of the revised version.**

please give the results special attention " Table 1 particularly"

**Answer. This has been addressed. The results section has been restructured into the following subheadings: “Descriptive analysis” and “Maternal sociodemographic determinants of child weight abnormalities”.**

**Table 1 has been updated with a more elaborated discussion. In addition, the dynamics of the key variables have been analysed and illustrated with a graph. Finally, a proper results interpretation has been provided under the sub heading “Maternal sociodemographic determinants of child weight abnormalities”**

**Please refer to pages 7, 8, 9, 10 of the revised version.**

as well as structure of the discussion must be conducted.

**Answer. The discussion section has been revised accordingly and this section further closes by the limitations of the study. It reads as follow:**

**The empirical findings reveal that maternal sociodemographic characteristics play a significant role in explaining child weight abnormalities in South Africa. Particularly, a positive association is found between maternal smoking and nutritional child health outcome, regardless of the proxy used. These results are in line with previous findings that children of smoking mothers are more likely to be overweight or obese than those of smoke-free mothers [10, 22]. This is understandable since smoking may lead to depression in mothers with overreaching consequences on child health either directly through unhealthy feeding scheme and nutritional choices or indirectly through genetic transfer. The genetic channel implies a transfer of parental weight abnormalities to their offspring; translating into a positive correlation between children weight and that of their parents. The results substantiate this assumption as mother’s hyper-obese status appears to be a significant driver of weight anomalies in children. Therefore, regular smoking mothers are at a greater risk of contributing to non-communicable diseases in their offspring. These non-communicable diseases include overweight or obesity, that is transferable from smoking parents, or being born from parents who suffer from overweight.**

**In addition, mothers' age, marital status and child grant recipient, display a consistent significant association with child health [23-24]. It is found that weight irregularities in children tend to increase with mothers’ age irrespective of the specification. This could probably indicate the inability to remain and/or follow a healthy life style as age increases with spillover effect on child care. It is also possible that age related weights gain in mothers can be transmitted to their children, namely those born by older mothers. Conversely, it is shown that children from married mothers are less exposed to weight problems compared to those from their unmarried counterparts. This finding could be attributed to the health benefit of a united family, including, reduced stress and anxiety, increased happiness and healthy life style, which boost the physical and emotional well-being [25]. Finally, child grant appears to reduce the likelihood of child weight problems. This finding suggests that child support programs can be a life-saving tool for children coming from poor households by releasing financial pressure to some extent, with positive influence on nutrition, which further improves child development. Notwithstanding the fact that living in urban areas expectedly offers better exposure to education and an awareness of malnutrition, with a possible reduction in the probability of being overweight and providing better care to offspring, “Urban” variable depicts a marginal decrease in the probability of children to be obese. Similarly, being Asian/Indian is significantly associated with the negative probability of the risk of being overweight in children with not on child overweight and obesity. This suggests a rather marginal racial difference in the prevalence of childhood overweight in South Africa.**

**However, the above inference is subject to the issue of attrition prevalent in dynamic surveys like NIDS dataset, which is attributed to the respondents’ relocation, death or non-responses. Moreover, because of data restrictions, the empirical strategy could not control for diet, which is claimed to have a significant influence on health challenges in children. The use of alternative data sources as they become available, could help mitigate these issues while providing further insight on the prevalence of childhood obesity in South Africa.**

Please ensure that your manuscript meets PLOS ONE's style requirements, including those for file naming.

**The references style has been updated consistently with the journal formatting requirements and the following declarations have been added after the conclusion:**

**Funding disclosure**

**No funding was received for this project.**

**Ethical statement**

**Ethics Approval for data collection for the five waves was granted by the University of Cape Town's Commerce Faculty Ethics in Research Committee on 12 December 2007 with additional approval for wave 5 granted by the Faculty of Health Sciences Human Research Ethics Committee on 19 December 2016. All the datasets are available in the public domain, and all information about respondent identities, used to track participants over time, has been excluded from the public release data.**

**For all the waves, the survey paper consent forms are issued in all official languages during the interviews and the informed consent process is conducted in the respondent's language of choice. The consent forms for children and young adults (15-17 years old) are signed by their caregivers. This was further supplemented in wave 5 by the verbal consent of the proxy respondents to allow the interview to be conducted on their behalf. In addition, the assent forms for children anthropometric measures require children aged between 7 to 10 years old to indicate their willingness to be measured. The interviews with missing signed consent forms are excluded from the dataset [26]**

**Author statement**

**Conceptualization and writing of the original draft:** N.Y. Nkomo

**Methodology, results interpretation/discussion, review and editing:** Prof B.D Simo-Kengne

**Results Interpretation and review:** Dr M. Biyase

**Declaration of competing interest**

There are no potential conflicts of interest.

**Reviewer 1**

- Title - Impact of maternal tobacco smoking on childhood obesity in South Africa - suggestion: Maternal tobacco smoking and childhood obesity: a cohort study

**Answer. The title has been updated accordingly. Thank you.**

abstract:
- review purpose - this study examines the affiliation... I would suggest to use the word association.

**Answer. The purpose component of the abstract has been updated to “Background” as per journal formatting requirement and the suggestion comment effected. It now reads:**

**Tobacco use and obesity are major public health problems and part of the leading causes of preventable disease and death worldwide. The prevalence of overweight children has escalated over the years; making the weight gain in children a critical issue for health professionals and academics alike. This study examines the association between maternal tobacco use and body weight abnormalities among South African children.**

- review Methods: "The link between maternal tobacco consumption and child health was assessed using logistic regression models in which unobserved child characteristics are assumed to be uncorrelated with independent variables. This random effect assumption allows us to control for time-invariant sociodemographic factors in estimating the probability of overweight prevalence in children. " - it does not describe the study design, variables studied, sample size calculation, inclusion and exclusion criteria.

**Answer. The method section of the abstract has been updated and now reads**

**The study uses data from the National Income Dynamics Study (NIDS), conducted from 2008 to 2017. The survey is available in five waves which have been merged into a panel dataset of 211,718 children aged between 0 to 5 years old, drawn from a nationally representative sample of households followed across time. Thus, the subgroup of children aged between 6 and 15 years old was excluded from the analysis. The empirical investigation employs a logistic regression model to estimate the marginal effect of maternal smoking on childhood overweight prevalence measured by three anthropometric based proxies, namely risk of child overweight, child overweight and child obesity. This framework assumes unobserved child characteristics to be uncorrelated with independent variables (random effect assumption); hence allowing to control for time-invariant sociodemographic factors which are likely to affect child nutritional health.**

- The results suggest... this is not appropriate to use the term suggest.

**Answer. This has been addressed throughout the text. It reads:**

**In addition to sociodemographic and health characteristics of mothers, empirical findings suggest that maternal smoking exhibits a significant odds and/ or probability of weight abnormalities in children. Notably, children of smoking mothers are likely to be obese, overweight, or at risk of becoming overweight with a possible coexistence of mother-child overweight. Similarly, weight irregularities in children are likely to increase with mothers’ age. Conversely, married mothers although associated with increased odds of children having high BMI, display a reduced probability of children being abnormally weighted. Finally, child support grant has the potential to improve children nutritional health as children whose mothers are recipient of such grant have a negative probability of having high BMI.**

- Introduction is fine, although the last sentence that one would expect the objective: "Therefore, understanding the contributing factors to childhood obesity becomes imperative in addressing overweight-related future public health challenges in South Africa." - it is not very straight forward as an objective - it should be reviewed.

**Answer. This has been addressed. It now reads as follow:**

**Accordingly, understanding the contributing factors to childhood obesity becomes imperative in addressing overweight-related future public health challenges in South Africa. Building on existing literature, this study investigates the possible association between maternal smoking and child weight abnormalities in south Africa using a panel logistic regression model.**

- There are some essential aspects that should be covered in the methods section.

**Answer. The method section has been updated to “Materials and Methods” with the sub-headings “Data” and “Empirical strategy” and their respective contents updated accordingly. “Data” provides details on the sampling, the variables construction and the ethical statement is further supplemented after the conclusion section as per the Journal requirements.**

**“The empirical strategy section” discusses the research design and details on the statistical analysis.**

**Please refer to pages 5 and 6 of the revised version.**

- The description of the result section are very superficial and do not cover the most relevant results

**Answer. This has been addressed. The estimation results have been structured into two sub sections, namely “descriptive analysis” and “Maternal sociodemographic determinants of child weight abnormalities”. Please refer to pages 5 and 6 of the revised version.**

**3.1. Descriptive analysis**

**Descriptive statistics show that approximately 82 percent children in the sample are at risk of being overweight, 76 percent are overweight and 74 percent are obese across the sample period. While the sampled children are gender balanced, the racial representation is 79 percent African, 13 percent Coloured, 1 percent Indian, and 4 percent White. In terms of maternal characteristics, approximately 48 percent of mothers live in urban areas, 90 percent of whom are overweight, 82 percent are obese, and 75 percent are hyper-obese. Mothers smoke on average a minimum of seven cigarettes daily and about 44 percent of them are married. In terms of the hypothesized correlation, Figure 1 depicts similar trends in child nutritional health measures and mothers’ smoking frequency across time. Consistently with maternal smoking, risk of obesity and obesity in children appear to have experienced a similar jump between wave 1 and wave 2, followed by a decreasing trend from wave 2 to wave 4. In the most recent wave, these dynamics have reversed with the rise of maternal smoking frequency as well as the increase fraction of overweight children although the percentage of children with the risk of overweight and the proportion of obese children have remained** constant.

**3.2. Maternal sociodemographic determinants of child weight abnormalities**

**The estimates of the effect of maternal smoking on each of the three child overweight prevalence proxies are summarized in Tables 2 and 3 where the dependent variable is respectively, risk of child overweight (Model 1), child overweight (Model 2) and child obese (Model 3). Each of the models regresses a child nutritional health proxy on maternal smoking, adjusting for control variables (child grant, the gender of the child, child birthweight, age of the mother, geographical areas, marital status of the mother, household size, father overweight, mother’s weight categories and different race groups).**

**Table 2 displays the estimated odds ratio with random effects, which allow to control for unobserved heterogeneity while accommodating for time invariant variables such as race. Across the three models, maternal smoking is significantly associated with an increased risk of child overweight (odds ratio 0.02), child overweight (odds ratio 0.02) and child obese (odds ratio 0.02). Expectedly, the results also reveal that child nutritional health significantly exhibits a positive odds with fathers overweight and mother obesity but a negative odds with mothers overweight. However, because the odds ratio bears less meaning with regard to the estimated probability, marginal effects displayed in Table 3 are provided for inference. Consistently with the odds ratio, maternal smoking has a positive association with child weight abnormalities. One extra cigarette smoked daily by a mother raises the odds of the risk of overweight, overweight and obesity in children by 0.0031, 0.0036 and 0.0042 basis points, respectively (Model 1, Model 2 and Model 3). These findings confirm the proposition that parental life style plays an important role in explaining offspring health at their early stage of life. Besides maternal smoking, mothers' characteristics, including age, marital status, and weight abnormalities, display a consistent significant association with child having high BMIs. Mother’s age and maternal hyperobesity status increase the probability of weight irregularities in children while being married has a mitigating effect on the likelihood of obesity, overweight and risk of overweight in children. Unsurprisingly, household size decreases the probability of weight abnormalities of children given the competing nutritional needs of children associated with a reduced risk of overfeeding as household size increases. Similarly, children with low birth weight rate are less likely to experience odd weight. In terms of population group, race does indeed have an impact on the odds of child weight issues, but only on the risk of overweight in children (Model 1), not on overweight (Model 2), or obesity (Model 3). In particular, being Asian/Indian reduces the likelihood of the child overweight risk, which might be attributed to their relatively healthy eating behaviour. Important to note also is the fact that living in urban areas and receiving a child grant are associated with a decreased odds and/or probability of weight abnormalities in children, yet with a generally weak association and/or inconsistently across the model.**

- The first sentence of the discussion is not well presented as you would expect that this sentence should be the main result of the study: "Because the odd ratio bears less meaning with regard to the estimated probability, marginal effects displayed in Table 3 are used for inference."

**Answer. This has been addressed. Please refer to the answers to the previous comments.**

- This sentence is not clearly presented: "The outcome of the study shows that, as the frequency of
maternal smoking increases, the likelihood for children to have abnormal weight also increases." - it has to be reviewed.

**Answer. This has been addressed. Please refer to the answers to the Updated results section.**

- The discussion is very superficial and needs to compare the results of the present study with the literature.

**Answer. This has been addressed. The revised discussion section reads:**

**4. Discussion**

**The empirical findings reveal that maternal sociodemographic characteristics play a significant role in explaining child weight abnormalities in South Africa. Particularly, a positive association is found between maternal smoking and nutritional child health outcome, regardless of the proxy used. These results are in line with previous findings that children of smoking mothers are more likely to be overweight or obese than those of smoke-free mothers [10, 22]. This is understandable since smoking may lead to depression in mothers with overreaching consequences on child health either directly through unhealthy feeding scheme and nutritional choices or indirectly through genetic transfer. The genetic channel implies a transfer of parental weight abnormalities to their offspring; translating into a positive correlation between children weight and that of their parents. The results substantiate this assumption as mother’s hyper-obese status appears to be a significant driver of weight anomalies in children. Therefore, regular smoking mothers are at a greater risk of contributing to non-communicable diseases in their offspring. These non-communicable diseases include overweight or obesity, that is transferable from smoking parents, or being born from parents who suffer from overweight.**

**In addition, mothers' age, marital status and child grant recipient, display a consistent significant association with child health [23-24]. It is found that weight irregularities in children tend to increase with mothers’ age irrespective of the specification. This could probably indicate the inability to remain and/or follow a healthy life style as age increases with spillover effect on child care. It is also possible that age related weights gain in mothers can be transmitted to their children, namely those born by older mothers. Conversely, it is shown that children from married mothers are less exposed to weight problems compared to those from their unmarried counterparts. This finding could be attributed to the health benefit of a united family, including, reduced stress and anxiety, increased happiness and healthy life style, which boost the physical and emotional well-being [25]. Finally, child grant appears to reduce the likelihood of child weight problems. This finding suggests that child support programs can be a life-saving tool for children coming from poor households by releasing financial pressure to some extent, with positive influence on nutrition, which further improves child development. Notwithstanding the fact that living in urban areas expectedly offers better exposure to education and an awareness of malnutrition, with a possible reduction in the probability of being overweight and providing better care to offspring, “Urban” variable depicts a marginal decrease in the probability of children to be obese. Similarly, being Asian/Indian is significantly associated with the negative probability of the risk of being overweight in children with not on child overweight and obesity. This suggests a rather marginal racial difference in the prevalence of childhood overweight in South Africa.**

- No limitation of the study has been presented.

**Answer. This has been added to in the discussion section and reads:**

**However, the above inference is subject to the issue of attrition prevalent in dynamic surveys like NIDS dataset, which is attributed to the respondents’ relocation, death or non-responses. Moreover, because of data restrictions, the empirical strategy could not control for diet, which is claimed to have a significant influence on health challenges in children. The use of alternative data sources as they become available, could help mitigate these issues while providing further insight on the prevalence of childhood obesity in South Africa.**

**Reviewer 2**

Reviewer #2: Dear authors’ thank you for submitting this nice manuscript.
Here under some comments about your manuscript.

**Answer. Thank you**

1. Try to summarize and minimize your manuscript introduction part.

**Answer. This has been addressed. Please refer to pages 3- 6 of the revised version.**

2. Write your study design clearly.

**Answer. This has been addressed. The method section has now been restructured into “Data” and “empirical strategy” subsections. The first subsection describes the sampling and variable construction while the latter focuses on the research design.**

3. Your dependent and independent variables are not clearly stated. List them.

**Answer. This has been addressed in the second paragraph of the “Data” subsection of the “Materials and Methods” section. It reads:**

**Three different child nutritional health indicators are used as dependent variable, namely child risk of being overweight, child overweight, and child obesity. These child health variables were created using the Body Mass Index (BMI) z-scores, which measure the relative weight that is adjusted for the age and gender of the child [21]. Accordingly, a child with a BMI z-score > 1, BMI z-score > 2 and BMI z-score > 3 is classified respectively as a risk to be overweight child, an overweight child and an obese child. The primary explanatory variable is mother smoking frequency (*M_smoking*), measured as the number of cigarettes smoked by a mother daily. The NIDS records each individual's height, weight, and waist measurement in the database, allowing us to construct the BMI using the weight (in kg) and the height in meters squared (m^2^). The study controls for the mothers' weight categorised into average weight, overweight, obese, and hyper obese, following the World Health Organisation index [21]. A person is classified as overweight if their BMI lies between 25 and 30, obese if BMI > 30, and hyper-obese if BMI > 35. South Africa is a culturally diverse country comprising four racial categories (African, White, Coloured, and Asian/Indian) with huge socioeconomic differences. Because of the high heterogeneity between South Africans and consistent with previous studies, the analysis controls for cultural, sociodemographic and economic characteristics of households. These include child grant recipient (C_grant), the gender of the child (Boy), child birthweight (C_birthweight), age of the mother (M_age), geographical areas (Urban), marital status of the mother (M_maritalstatus), household size (Hhsize), father overweight (F_overweight), mother’s weight (M_overweight, M_obese, M_hyperobese, Average), and race (White, Asian/Indian, Coloured and Black). The details on variables definitions and summary statistics are provided in Table 1**

4. What is the meaning of Table 1??? It is meaningless, because it is known that computing the mean, sd, minimum and maximum values for categorical variables are impossible. So remove this table, unless revise it.

**Answer. This has been addressed. Table 1 has been updated accordingly. Please see below the revised table.**

**Table 1. Descriptive statistics**

| **Variables** | **Description of variables** | **Mean/Frequency** | **Std. Dev.** |
| --- | --- | --- | --- |
| ***Dependent Variables*** |  |  |  |
| **Child Health** |  |  |  |
| **Risk of Child Overweight** | **BMI-for-age Z-score > 1.0** | **.82** | **.37** |
| **Child Overweight** | **BMI-for-age Z-score >2.0** | **.76** | **.42** |
| **Child Obese** | **BMI-for-age Z-score >3.0** | **.74** | **.43** |
| ***Explanatory variable*** |  |  |  |
| **Smoking Variable** |  |  |  |
| **Maternal Smoking** | **Number of cigarettes smoked per day** | **7.70** | **6.44** |
| **Maternal characteristics** |  |  |  |
| **Maternal age** | **Age of the mother (continuous variable)** | **42.36** | **14.14** |
| **Married mother** | **Married mother(Yes=1)** | **0.44** | **0.49** |
| **Maternal overweight** | **BMI_mother > = 25** | **.90** | **.28** |
| **Maternal obese** | **BMI_mother > = 30** | **.82** | **.37** |
| **Maternal hyperobese** | **BMI_mother > = 35** | **.75** | **.43** |
| **Covariates of outcome model** |  |  |  |
| **Child gender** | **Boy (Yes=1)** | **.50** | **.50** |
| **Urban** | **Urban (Yes=1)** | **.48** | **.49** |
| **Child birth weight** | **Birth weight of child (continuous)** | **3.33** | **9.58** |
| **African** | **Race category 1 (Yes =1)** | **.79** | **.40** |
| **Coloured** | **Race category 2 (Yes =1)** | **.13** | **.34** |
| **Indian** | **Race category 3 (Yes =1)** | **.01** | **.12** |
| **White** | **Race category 4 (Yes =1)** | **.04** | **.21** |
| **Father overweight** | **BMI_mother > = 25** | **.97** | **.15** |

5. Your results in the table 2 and table 3 are not correctly presented. I think that it is meaningless. Where is the estimates of the parameters? Where is the interpretation of the parameters?

**Answer. The results section has been structure and detailed interpretation of tables 2 and 3 provided in the second sub section as follows:**

**3.2. Maternal sociodemographic determinants of child weight abnormalities**

**The estimates of the effect of maternal smoking on each of the three child health proxies are summarized in Tables 2 and 3 where the dependent variable is respectively, risk of child overweight (Model 1), child overweight (Model 2) and child obese (Model 3). Each of the models regresses a child nutritional health proxy on maternal smoking, adjusting for control variables (child grant, the gender of the child, child birthweight, age of the mother, geographical areas, marital status of the mother, household size, father overweight, mother’s weight categories and different race groups).**

**Table 2 displays the estimated odds ratio with random effects, which allow to control for unobserved heterogeneity while accommodating for time invariant variables such as race. Across the three models, maternal smoking is significantly associated with an increased risk of child overweight (odds ratio 0.02), child overweight (odds ratio 0.02) and child obese (odds ratio 0.02). Expectedly, the results also reveal that child nutritional health significantly exhibits a positive odds with fathers overweight and mother obesity but a negative odds with mothers overweight. However, because the odds ratio bears less meaning with regard to the estimated probability, marginal effects displayed in Table 3 are provided for inference. Consistently with the odds ratio, maternal smoking has a positive association with child weight abnormalities. One extra cigarette smoked daily by a mother raises the odds of the risk of overweight, overweight and obesity in children by 0.0031, 0.0036 and 0.0042 basis points, respectively (Model 1, Model 2 and Model 3). These findings confirm the proposition that parental life style plays an important role in explaining offspring health at their early stage of life. Besides maternal smoking, mothers' characteristics, including age, marital status, and weight abnormalities, display a consistent significant association with child having high BMIs. Mother’s age and maternal hyperobesity status increase the probability of weight irregularities in children while being married has a mitigating effect on the likelihood of obesity, overweight and risk of overweight in children. Unsurprisingly, household size decreases the probability of weight abnormalities of children given the competing nutritional needs of children associated with a reduced risk of overfeeding as household size increases. Similarly, children with low birth weight rate are less likely to experience odd weight. In terms of population group, race does indeed have an impact on the odds of child weight issues, but only on the risk of overweight in children (Model 1), not on overweight (Model 2), or obesity (Model 3). In particular, being Asian/Indian reduces the likelihood of the child overweight risk, which might be attributed to their relatively healthy eating behaviour. Important to note also is the fact that living in urban areas and receiving a child grant are associated with a decreased odds and/or probability of weight abnormalities in children, yet with a generally weak association and/or inconsistently across the model.**

6. General try to re-write your manuscript.

**Answer. This has been addressed. Thank you**

**Reviewer 3**

Reviewer #3: Dear author, I attached some of my comments here.
-_You have to put line number for the whole part of your manuscript

**Answer. This has been addressed. The revised version of the manuscripts includes the line numbers.**

Abstract
-You did not included introduction.

**Answer.This has been addressed under “background” as follows:**

**Tobacco use and obesity are major public health problems and part of the leading causes of preventable disease and death worldwide. The prevalence of overweight children has escalated over the years; making the weight gain in children a critical issue for health professionals and academics alike. This study examines the association between maternal tobacco use and body weight abnormalities among South African children.**

Abstract
-The method part does not include important information like Sample size, sampling technique, study design, the major assumptions checked and the measures of association used.

**Answer. This has been addressed. The updated “method” section of the abstract reads:**

**The study uses data from the National Income Dynamics Study (NIDS), conducted from 2008 to 2017. The survey is available in five waves which have been merged into a panel dataset of 211,718 children aged between 0 to 5 years old, drawn from a nationally representative sample of households followed across time. Thus, the subgroup of children aged between 6 and 15 years old was excluded from the analysis. The empirical investigation employs a logistic regression model to estimate the marginal effect of maternal smoking on childhood overweight prevalence measured by three anthropometric based proxies, namely risk of child overweight, child overweight and child obesity. This framework assumes unobserved child characteristics to be uncorrelated with independent variables (random effect assumption); hence allowing to control for time-invariant sociodemographic factors which are likely to affect child nutritional health.**

Methods
-Why you put all portions of the method in one paragraph. It is better to put in different sub-headings.

**Answer. This has been addressed. The method section has been updated to “Materials and Methods” with the sub-headings “Data” and “Empirical strategy” and their respective contents updated accordingly. Please refer to pages 5 and 6 of the revised version.**

Methods
-Although it is secondary data, for the reader of the paper to be clear you have to elaborate about the sampling method, the way you reach the sample size, data collection tools and procedures.

**Answer. This has been addressed under “Data” subsection of the “Materials and Methods” section as follows:**

**2.1 Data**

**This study uses the National Income Dynamics Study (NIDS) survey, a longitudinal household study in South Africa conducted by the Southern Africa Labour and Development Research Unit (SALDRU) based at the University of Cape Town’s School of Economics. Started in 2008, NIDS is repeated every two years by tracking a nationally representative sample of the same households across the country to collect information on people livelihood using (i) the households questionnaire (on household characteristics, household roster, mortality history, living standards, expenditure, consumption, negative events, positive events, agriculture), (ii) the adults questionnaire (on demographics, education, labour market participation, income, health, well-being, numeracy, anthropometric data) and (iii) the children questionnaire (on education, health, family support, grants, anthropometric data, numeracy).**

**To investigate the association between maternal smoking and child health, a cohort study is performed on children aged between 0 to 5 years old over the period from 2008 to 2017. Since children are defined in South Africa as persons below the age of 15, the subgroup of children aged between 6 and 15 years old was excluded from the analysis. The survey is available in five waves which have been merged into a panel dataset of 62 243 children arranged according to child person identifier. Three different child nutritional health indicators are used as dependent variable, namely child risk of being overweight, child overweight, and child obesity. These child health variables were created using the Body Mass Index (BMI) z-scores, which measure the relative weight that is adjusted for the age and gender of the child [21]. Accordingly, a child with a BMI z-score > 1, BMI z-score > 2 and BMI z-score > 3 is classified respectively as a risk to be overweight child, an overweight child and an obese child. The primary explanatory variable is mother smoking frequency (*M_smoking*), measured as the number of cigarettes smoked by a mother daily. The NIDS records each individual's height, weight, and waist measurement in the database, allowing us to construct the BMI using the weight (in kg) and the height in meters squared (m^2^). The study controls for the mothers' weight categorised into average weight, overweight, obese, and hyper obese, following the World Health Organisation index [21]. A person is classified as overweight if their BMI lies between 25 and 30, obese if BMI > 30, and hyper-obese if BMI > 35.**

**South Africa is a culturally diverse country comprising four racial categories (African, White, Coloured, and Asian/Indian) with huge socioeconomic differences. Because of the high heterogeneity between South Africans and consistent with previous studies, the analysis controls for cultural, sociodemographic and economic characteristics of households. These include child grant recipient (*C_grant*), the gender of the child (*Boy*), child birthweight (*C_birthweight*), age of the mother (*M_age*), geographical areas (*Urban*), marital status of the mother (*M_maritalstatus*), household size (*Hhsize*), father overweight (*F_overweight*), mother’s weight (*M_overweight*, *M_obese*, *M_hyperobese*, *Average*), and race (*White*, *Asian/Indian*, *Coloured* and *Black*). The details on variables definitions and summary statistics are provided in Table 1.**

Methods
-What about the inclusion and exclusion criteria, data quality control and ethical assurance?

**Answer. This has been addressed. The inclusion/exclusion criteria are discussed under “Data” sub section and the ethical statement has been added after the conclusion section as per the journal requirements. It reads:**

**Ethical statement**

**Ethics Approval for data collection for the five waves was granted by the University of Cape Town's Commerce Faculty Ethics in Research Committee on 12 December 2007 with additional approval for wave 5 granted by the Faculty of Health Sciences Human Research Ethics Committee on 19 December 2016. All the datasets are available in the public domain, and all information about respondent identities, used to track participants over time, has been excluded from the public release data.**

**For all the waves, the survey paper consent forms are issued in all official languages during the interviews and the informed consent process is conducted in the respondent's language of choice. The consent forms for children and young adults (15-17 years old) are signed by their caregivers. This was further supplemented in wave 5 by the verbal consent of the proxy respondents to allow the interview to be conducted on their behalf. In addition, the assent forms for children anthropometric measures require children aged between 7 to 10 years old to indicate their willingness to be measured. The interviews with missing signed consent forms are excluded from the dataset [26]**

Methods
-You didn’t described about the models (what is included in model1, model2 and model3 and what criteria was used to compare those models and which one is better)

**Answer. This has ben addressed under the second subsection of the “Results” section. It reads:**

**The estimates of the effect of maternal smoking on each of the three child overweight prevalence proxies are summarized in Tables 2 and 3 where the dependent variable is respectively, risk of child overweight (Model 1), child overweight (Model 2) and child obese (Model 3). Each of the models regresses a child overweight prevalence proxy on maternal smoking, adjusting for control variables (child grant, the gender of the child, child birthweight, age of the mother, geographical areas, marital status of the mother, household size, father overweight, mother’s weight categories and different race groups).**

Methods
-What measure of association you had used?

**Answer. Regression estimates from the logistic model (odds ratio and marginal effect) were used as the measures of association.**

Result
-Why did you prefer using mean and standard deviation?

**Answer. The summary statistics were provided to describe the data used. We understand that the discussion was scanty and quasi unrelated with the objective to the study. The revised manuscript now motivates the need to have a summary statistics by adding the descriptive analysis to prior to the inferential investigation.**

Result
-The variables did not have category

**Answer. It was difficult to tell from the earlier version of the manuscript whether there are categorical variables or not. We have not only updated the data and variable description, but the summary statistics table has been revised to include the definition of each variable used, from which categorical variables can be identified from the continuous ones.**

Result
-The tables need full title, which describes about place, person, time and the problem. In addition, above the table you have to describe major things from the table using texts.

**Answer. All the tables/Figure have updated headings with explicit description of what the tables are about (see below). In addition, notes are provided below the Tables, explaining what the displayed figures represent and how to interpret symbols.**

**Table 1: Descriptive statistics of variables used in the estimation**

**Figure 1. Trend in childhood overweight prevalence and maternal smoking across waves**

**Table 2. Odds of maternal smoking on childhood overweight prevalence**

**Table 3: Marginal effects of maternal smoking on childhood overweight prevalence**

Result
- In result section, Interpretation of the finding is mandatory

**Answer. The “Results” section has been restructured into a preliminary analysis and the inferential analysis sub sections named “Descriptive analysis” and “Maternal sociodemographic determinants of child weight abnormalities”, respectively. The latter provides the interpretation of the estimation output as follows:**

**3.2. Maternal sociodemographic determinants of child weight abnormalities**

**The estimates of the effect of maternal smoking on each of the three child overweight prevalence proxies are summarized in Tables 2 and 3 where the dependent variable is respectively, risk of child overweight (Model 1), child overweight (Model 2) and child obese (Model 3). Each of the models regresses a child nutritional health proxy on maternal smoking, adjusting for control variables (child grant, the gender of the child, child birthweight, age of the mother, geographical areas, marital status of the mother, household size, father overweight, mother’s weight categories and different race groups).**

**Table 2 displays the estimated odds ratio with random effects, which allow to control for unobserved heterogeneity while accommodating for time invariant variables such as race. Across the three models, maternal smoking is significantly associated with an increased risk of child overweight (odds ratio 0.02), child overweight (odds ratio 0.02) and child obese (odds ratio 0.02). Expectedly, the results also reveal that child nutritional health significantly exhibits a positive odds with fathers overweight and mother obesity but a negative odds with mothers overweight. However, because the odds ratio bears less meaning with regard to the estimated probability, marginal effects displayed in Table 3 are provided for inference. Consistently with the odds ratio, maternal smoking has a positive association with child weight abnormalities. One extra cigarette smoked daily by a mother raises the odds of the risk of overweight, overweight and obesity in children by 0.0031, 0.0036 and 0.0042 basis points, respectively (Model 1, Model 2 and Model 3). These findings confirm the proposition that parental life style plays an important role in explaining offspring health at their early stage of life. Besides maternal smoking, mothers' characteristics, including age, marital status, and weight abnormalities, display a consistent significant association with child having high BMIs. Mother’s age and maternal hyperobesity status increase the probability of weight irregularities in children while being married has a mitigating effect on the likelihood of obesity, overweight and risk of overweight in children. Unsurprisingly, household size decreases the probability of weight abnormalities of children given the competing nutritional needs of children associated with a reduced risk of overfeeding as household size increases. Similarly, children with low birth weight rate are less likely to experience odd weight. In terms of population group, race does indeed have an impact on the odds of child weight issues, but only on the risk of overweight in children (Model 1), not on overweight (Model 2), or obesity (Model 3). In particular, being Asian/Indian reduces the likelihood of the child overweight risk, which might be attributed to their relatively healthy eating behaviour. Important to note also is the fact that living in urban areas and receiving a child grant are associated with a decreased odds and/or probability of weight abnormalities in children, yet with a generally weak association and/or inconsistently across the model.**

Discussion
-you did not included the possible justifications

**Answer. This has been addressed. The revised discussion section reads:**

**The empirical findings reveal that maternal sociodemographic characteristics play a significant role in explaining child weight abnormalities in South Africa. Particularly, a positive association is found between maternal smoking and nutritional child health outcome, regardless of the proxy used. These results are in line with previous findings that children of smoking mothers are more likely to be overweight or obese than those of smoke-free mothers [10, 22]. This is understandable since smoking may lead to depression in mothers with overreaching consequences on child health either directly through unhealthy feeding scheme and nutritional choices or indirectly through genetic transfer. The genetic channel implies a transfer of parental weight abnormalities to their offspring; translating into a positive correlation between children weight and that of their parents. The results substantiate this assumption as mother’s hyper-obese status appears to be a significant driver of weight anomalies in children. Therefore, regular smoking mothers are at a greater risk of contributing to non-communicable diseases in their offspring. These non-communicable diseases include overweight or obesity, that is transferable from smoking parents, or being born from parents who suffer from overweight.**

**In addition, mothers' age, marital status and child grant recipient, display a consistent significant association with child health [23-24]. It is found that weight irregularities in children tend to increase with mothers’ age irrespective of the specification. This could probably indicate the inability to remain and/or follow a healthy life style as age increases with spillover effect on child care. It is also possible that age related weights gain in mothers can be transmitted to their children, namely those born by older mothers. Conversely, it is shown that children from married mothers are less exposed to weight problems compared to those from their unmarried counterparts. This finding could be attributed to the health benefit of a united family, including, reduced stress and anxiety, increased happiness and healthy life style, which boost the physical and emotional well-being [25]. Finally, child grant appears to reduce the likelihood of child weight problems. This finding suggests that child support programs can be a life-saving tool for children coming from poor households by releasing financial pressure to some extent, with positive influence on nutrition, which further improves child development. Notwithstanding the fact that living in urban areas expectedly offers better exposure to education and an awareness of malnutrition, with a possible reduction in the probability of being overweight and providing better care to offspring, “Urban” variable depicts a marginal decrease in the probability of children to be obese. Similarly, being Asian/Indian is significantly associated with the negative probability of the risk of being overweight in children with not on child overweight and obesity. This suggests a rather marginal racial difference in the prevalence of childhood overweight in South Africa.**

**Reviewer 4**

Reviewer #4: The authors conducted a large-scale study examining the association between maternal tobacco use and children's health in South Africa in terms of body weight condition. In general terms the article is clearly stated, well documented, easy to follow.
However, there are some minor issues that the authors could address:

**Answer. Thank you**

-The introduction presents the scientific background very explanatory, but I think it is necessary that the authors state more clearly in this section the specific objectives and hypotheses.

**Answer. The introduction has been revised and the objective and hypotheses explicitly formulated. Please refer to pages 3 to 6 of the revised version. Particularly, the last paragraph of pages 5 and 6 reads:**

**Last Paragraph page 5, lines 136 and 137 reads:**

**Considering the high level of smoking and overweight challenges in South Africa [6], this study hypothesizes and tests the overweight children's likelihood to have regular smoking mothers.**

**Last paragraph Page 6, lines 167 and 171 reads:**

**Accordingly, understanding the contributing factors to childhood obesity becomes imperative in addressing overweight-related future public health challenges in South Africa. Building on existing literature, this study investigates the possible association between maternal smoking and child weight abnormalities in south Africa using a panel logistic regression model.**

-I suggest that to reduce the ambiguity of this paper, the authors should present the methods in a structured way, in line with the STROBE Statement for observational studies. Also, in the methods section the authors should present the study design, settings, and participant eligibility criteria. These seem to be missing or are presented ambiguously.

**Answer. This has been addressed under “Materials and Methods” section, which is further structured into two subsections, namely “Data” and “Empirical strategy”. Unlike the empirical strategy subsection which requires minor revision, major attention was given to the data subsection, which now includes all the suggested missing details. This is further supplemented by the ethical clearance located after the conclusion section consistently to the journal formatting requirement. It reads as follows:**

**2.1 Data**

**This study uses the National Income Dynamics Study (NIDS) survey, a longitudinal household study in South Africa conducted by the Southern Africa Labour and Development Research Unit (SALDRU) based at the University of Cape Town’s School of Economics. Started in 2008, NIDS is repeated every two years by tracking a nationally representative sample of the same households across the country to collect information on people livelihood using (i) the households questionnaire (on household characteristics, household roster, mortality history, living standards, expenditure, consumption, negative events, positive events, agriculture), (ii) the adults questionnaire (on demographics, education, labour market participation, income, health, well-being, numeracy, anthropometric data) and (iii) the children questionnaire (on education, health, family support, grants, anthropometric data, numeracy).**

**To investigate the association between maternal smoking and child health, a cohort study is performed on children aged between 0 to 5 years old over the period from 2008 to 2017. Since children are defined in South Africa as persons below the age of 15, the subgroup of children aged between 6 and 15 years old was excluded from the analysis. The survey is available in five waves which have been merged into a panel dataset of 62 243 children arranged according to child person identifier. Three different child nutritional health indicators are used as dependent variable, namely child risk of being overweight, child overweight, and child obesity. These child health variables were created using the Body Mass Index (BMI) z-scores, which measure the relative weight that is adjusted for the age and gender of the child [21]. Accordingly, a child with a BMI z-score > 1, BMI z-score > 2 and BMI z-score > 3 is classified respectively as a risk to be overweight child, an overweight child and an obese child. The primary explanatory variable is mother smoking frequency (*M_smoking*), measured as the number of cigarettes smoked by a mother daily. The NIDS records each individual's height, weight, and waist measurement in the database, allowing us to construct the BMI using the weight (in kg) and the height in meters squared (m^2^). The study controls for the mothers' weight categorised into average weight, overweight, obese, and hyper obese, following the World Health Organisation index [21]. A person is classified as overweight if their BMI lies between 25 and 30, obese if BMI > 30, and hyper-obese if BMI > 35.**

**South Africa is a culturally diverse country comprising four racial categories (African, White, Coloured, and Asian/Indian) with huge socioeconomic differences. Because of the high heterogeneity between South Africans and consistent with previous studies, the analysis controls for cultural, sociodemographic and economic characteristics of households. These include child grant recipient (*C_grant*), the gender of the child (*Boy*), child birthweight (*C_birthweight*), age of the mother (*M_age*), geographical areas (*Urban*), marital status of the mother (*M_maritalstatus*), household size (*Hhsize*), father overweight (*F_overweight*), mother’s weight (*M_overweight*, *M_obese*, *M_hyperobese*, *Average*), and race (*White*, *Asian/Indian*, *Coloured* and *Black*). The details on variables definitions and summary statistics are provided in Table 1.**

**Ethical statement**

**Ethics Approval for data collection for the five waves was granted by the University of Cape Town's Commerce Faculty Ethics in Research Committee on 12 December 2007 with additional approval for wave 5 granted by the Faculty of Health Sciences Human Research Ethics Committee on 19 December 2016. All the datasets are available in the public domain, and all information about respondent identities, used to track participants over time, has been excluded from the public release data.**

**For all the waves, the survey paper consent forms are issued in all official languages during the interviews and the informed consent process is conducted in the respondent's language of choice. The consent forms for children and young adults (15-17 years old) are signed by their caregivers. This was further supplemented in wave 5 by the verbal consent of the proxy respondents to allow the interview to be conducted on their behalf. In addition, the assent forms for children anthropometric measures require children aged between 7 to 10 years old to indicate their willingness to be measured. The interviews with missing signed consent forms are excluded from the dataset [26]**

- In the results section, the descriptive statistics presented in Table 1 appear to be incorrect. The authors should present categorical variables as numbers (percentage, confidence interval).

**Answer. This has been addressed. The update summary statistics (Table 1) now includes the variable definitions column to identify the categorical variable. In addition, the frequency of the categorical variable is discussed in terms of percentage as follows:**

**Descriptive statistics show that approximately 82 percent children in the sample are at risk of being overweight, 76 percent are overweight and 74 percent are obese across the sample period. While the sampled children are gender balanced, the racial representation is 79 percent African, 13 percent Coloured, 1 percent Indian, and 4 percent White. In terms of maternal characteristics, approximately 48 percent of mothers live in urban areas, 90 percent of whom are overweight, 82 percent are obese, and 75 percent are hyper-obese. Mothers smoke on average a minimum of seven cigarettes daily and about 44 percent of them are married.**

-Regression models should also be explained in more detail, clearly showing unstandardized and standardized regression weights and p-values. Odds ratios could also be presented.

**Answer. This has been addressed under the second sub section of the results section. Please see below:**

**3.2. Maternal sociodemographic determinants of child weight abnormalities**

**The estimates of the effect of maternal smoking on each of the three child overweight prevalence proxies are summarized in Tables 2 and 3 where the dependent variable is respectively, risk of child overweight (Model 1), child overweight (Model 2) and child obese (Model 3). Each of the models regresses a child nutritional health proxy on maternal smoking, adjusting for control variables (child grant, the gender of the child, child birthweight, age of the mother, geographical areas, marital status of the mother, household size, father overweight, mother’s weight categories and different race groups).**

**Table 2 displays the estimated odds ratio with random effects, which allow to control for unobserved heterogeneity while accommodating for time invariant variables such as race. Across the three models, maternal smoking is significantly associated with an increased risk of child overweight (odds ratio 0.02), child overweight (odds ratio 0.02) and child obese (odds ratio 0.02). Expectedly, the results also reveal that child nutritional health significantly exhibits a positive odds with fathers overweight and mother obesity but a negative odds with mothers overweight. However, because the odds ratio bears less meaning with regard to the estimated probability, marginal effects displayed in Table 3 are provided for inference. Consistently with the odds ratio, maternal smoking has a positive association with child weight abnormalities. One extra cigarette smoked daily by a mother raises the odds of the risk of overweight, overweight and obesity in children by 0.0031, 0.0036 and 0.0042 basis points, respectively (Model 1, Model 2 and Model 3). These findings confirm the proposition that parental life style plays an important role in explaining offspring health at their early stage of life. Besides maternal smoking, mothers' characteristics, including age, marital status, and weight abnormalities, display a consistent significant association with child having high BMIs. Mother’s age and maternal hyperobesity status increase the probability of weight irregularities in children while being married has a mitigating effect on the likelihood of obesity, overweight and risk of overweight in children. Unsurprisingly, household size decreases the probability of weight abnormalities of children given the competing nutritional needs of children associated with a reduced risk of overfeeding as household size increases. Similarly, children with low birth weight rate are less likely to experience odd weight. In terms of population group, race does indeed have an impact on the odds of child weight issues, but only on the risk of overweight in children (Model 1), not on overweight (Model 2), or obesity (Model 3). In particular, being Asian/Indian reduces the likelihood of the child overweight risk, which might be attributed to their relatively healthy eating behaviour. Important to note also is the fact that living in urban areas and receiving a child grant are associated with a decreased odds and/or probability of weight abnormalities in children, yet with a generally weak association and/or inconsistently across the model.**

-The discussion summarizes the results, but I think the conclusions are very long. Much of that presented in the conclusions section should be moved to the discussion.

**Answer. This has been addressed. The updated “Discussion” and “Conclusion” sections read respectively as follow:**

**4. Discussion**

**The empirical findings reveal that maternal sociodemographic characteristics play a significant role in explaining child weight abnormalities in South Africa. Particularly, a positive association is found between maternal smoking and nutritional child health outcome, regardless of the proxy used. These results are in line with previous findings that children of smoking mothers are more likely to be overweight or obese than those of smoke-free mothers [10, 22]. This is understandable since smoking may lead to depression in mothers with overreaching consequences on child health either directly through unhealthy feeding scheme and nutritional choices or indirectly through genetic transfer. The genetic channel implies a transfer of parental weight abnormalities to their offspring; translating into a positive correlation between children weight and that of their parents. The results substantiate this assumption as mother’s hyper-obese status appears to be a significant driver of weight anomalies in children. Therefore, regular smoking mothers are at a greater risk of contributing to non-communicable diseases in their offspring. These non-communicable diseases include overweight or obesity, that is transferable from smoking parents, or being born from parents who suffer from overweight.**

**In addition, mothers' age, marital status and child grant recipient, display a consistent significant association with child health [23-24]. It is found that weight irregularities in children tend to increase with mothers’ age irrespective of the specification. This could probably indicate the inability to remain and/or follow a healthy life style as age increases with spillover effect on child care. It is also possible that age related weights gain in mothers can be transmitted to their children, namely those born by older mothers. Conversely, it is shown that children from married mothers are less exposed to weight problems compared to those from their unmarried counterparts. This finding could be attributed to the health benefit of a united family, including, reduced stress and anxiety, increased happiness and healthy life style, which boost the physical and emotional well-being [25]. Finally, child grant appears to reduce the likelihood of child weight problems. This finding suggests that child support programs can be a life-saving tool for children coming from poor households by releasing financial pressure to some extent, with positive influence on nutrition, which further improves child development. Notwithstanding the fact that living in urban areas expectedly offers better exposure to education and an awareness of malnutrition, with a possible reduction in the probability of being overweight and providing better care to offspring, “Urban” variable depicts a marginal decrease in the probability of children to be obese. Similarly, being Asian/Indian is significantly associated with the negative probability of the risk of being overweight in children with not on child overweight and obesity. This suggests a rather marginal racial difference in the prevalence of childhood overweight in South Africa.**

**However, the above inference is subject to the issue of attrition prevalent in dynamic surveys like NIDS dataset, which is attributed to the respondents’ relocation, death or non-responses. Moreover, because of data restrictions, the empirical strategy could not control for diet, which is claimed to have a significant influence on health challenges in children. The use of alternative data sources as they become available, could help mitigate these issues while providing further insight on the prevalence of childhood obesity in South Africa.**

**5. Conclusion**

**This study assesses the link between maternal smoking frequency (number of cigarettes smoked per day) and childhood weight status in South Africa, using a random-effect logic model implemented on a panel dataset made of the five NIDS waves. Overall, evidence shows that maternal smoking contributes to child overweight and/or obesity. In addition, the mother’s hyperobese status is a significant driver of weight anomalies in children, which motivates the importance of early interventions targeted at mothers. The results further indicate the influence of childhood issues and exposure to harsh early life factors on a child’s weight status.**

**Because of the complexity of overweight challenges, there is a need to strengthen population health policies linked to implementing educational programs to minimise tobacco consumption. Policymakers can prioritise programs to reduce smoking behaviour, especially amongst pregnant women and caregivers, to minimise the risk of childhood overweight. Furthermore, comprehensive action plans and recommendations that address norms and make treatment plans available for the children who are already suffering from overweight, are essential. These include child support programs, the promotion and the consumption of healthy foods accompanied by physical activity, which may reduce mothers' stress levels and their incentive to self-medicate using tobacco substances. Finally, Parents must be made aware of the different adverse outcomes of tobacco smoking as one of the most preventable causes of childhood overweight or obesity, among other child-related development complications.**
